# Data for labor market concentration using Lightcast (formerly Burning Glass Technologies)

**DOI:** 10.1016/j.dib.2024.110647

**Published:** 2024-06-18

**Authors:** Hyeri Choi, Ioana Marinescu

**Affiliations:** a School of Labor and Employment Relations, University of Illinois, Urbana-Champaign, 504 E. Armory Avenue, Champaign, IL 61820, United States; b School of Social Policy & Practice, University of Pennsylvania, 3701 Locust Walk, Philadelphia, PA 19104-6214, United States

**Keywords:** Labor market, Employer market power, Herfindahl-Hirschman Index, Labor demand, Job vacancies

## Abstract

This data article provides a description of the labor market concentration dataset. Using the job vacancy data from Lightcast from 2007Q1 to 2021Q2 (2008 and 2009 data are not available), we measure labor market concentration by using Herfindahl-Hirschman Index (HHI) in labor markets defined at the occupation (six-digit SOC), commuting zone, and quarterly level. The HHI is calculated based on the share of vacancies among all the firms that post vacancies in that market. Data includes information on year-quarter, six-digit SOC, commuting zone, lower bound HHI, and higher bound HHI. Given the growing literature on labor monopsony power, this labor market concentration data can be used by researchers in various contexts, aiming to investigate the impact of employer market power on different labor market and social outcomes.

Specifications Table

**Table:** 

Subject	Macroeconomics
Specific subject area	Labor economics
Data format	Analyzed data
Type of data	Stata (.dta.) file
Data collection	The original data were collected and cleaned by Lightcast in the U.S. from online job postings. The company collects and de-duplicates job postings from about 40,000 websites, which constitutes most of the U.S. job vacancies posted online.
Data source location	The original job posting data were collected and cleaned by Lightcast in the U.S., and the data are stored in a hard drive at the University of Pennsylvania. The supplemental data, county-commuting zone crosswalk, is available on the USDA website at https://www.ers.usda.gov/data-products/commuting-zones-and-labor-market-areas/
Data accessibility	Repository name: Mendeley Data identification number: doi: 10.17632/45r6ps4r68.1 Direct URL to data: https://data.mendeley.com/datasets/45r6ps4r68/1 Instructions for accessing these data: You can access the data by going to Mendeley using the above URL. Choi, Hyeri; Marinescu, Ioana (2023), “Data for labor market concentration using Lightcast (formerly Burning Glass Technologies)”, Mendeley Data, V1, doi: 10.17632/45r6ps4r68.1
Related research article	H**.** Choi, I. Marinescu, The labor demand side of involuntary part-time employment, Research in Labor Economics. In Press.

## 1 Value of the Data

• Researchers who are interested in examining the impact of monopsony power can benefit from using this data. With growing literature on employer market power, this labor market concentration data can be used by researchers in various contexts, aiming to investigate the impact of employer market power at the local labor market level.
• Researchers can integrate this dataset with other sources of information that provide data on geography, occupation, and time. This flexibility allows for comprehensive analyses by merging datasets and gaining a more holistic understanding of labor market dynamics and related social and economic phenomena.

## 2 Background

Monopsony power in the labor market leads to wage suppression and decreased employment [2,3]. An increasing number of researchers and policymakers are showing keen interest in a concentrated labor market which is characterized by a small number of employers dominating hiring. Analyzing job vacancy data is crucial for understanding labor market dynamics from the employer's perspective. While the Job Openings and Labor Turnover Survey (JOLTS) collects information from a nationally representative sample of employers about near-term vacancies, its data are typically limited to aggregated levels and lack detailed vacancy characteristics. Thus, we aim to enrich the understanding of labor market concentration by utilizing a more detailed dataset. In addition to the upcoming article by Choi and Marinescu [1], which calculated labor market concentration using census OCC, commuting zone, and year-quarter to align with the Current Population Survey, this dataset offers added value by employing six-digit SOC. This more detailed occupational classification provides deeper insights into local labor market dynamics.

## 3 Data Description

This article describes the labor market concentration dataset between 2007Q1 to 2021Q2. Data consists of variables on year-quarter, six-digit SOC, commuting zone (CZ), lower bound Herfindahl-Hirschman Index (HHI), and higher bound HHI [7]. The data is organized by commuting zone, SOC, and year-quarter. Calculating labor market concentration requires the Lightcast job postings data and crosswalks between counties and commuting zones. The original Lightcast dataset is proprietary, whereas the accompanying dataset is readily available in the Mendeley repository folder.

We use data on all online vacancies from Lightcast from 2007Q1 to 2021Q2 (2008 and 2009 data are not available). The company collects and de-duplicates job postings from about 40,000 websites, which constitutes most of the US job vacancies posted online. Lightcast only measures new postings, and vacancies posted on multiple sites are represented only once.

From Lightcast data, we measure the number of vacancies for each firm and identify the location and occupation of each vacancy. The differentiation of firms is established through the employer name. In the original Lightcast data, the employer variable has been standardized, grouping variants of employer names together. For instance, postings from “Burning Glass,” “Burning Glass Technologies,” and “Burning Glass International, Inc.” are all standardized to “Burning Glass Technologies.”

The Lightcast data provides geographic information including city, state, county, and metropolitan statistical area. To measure labor market concentration at the local labor market level, we use the county variable to match with commuting zones. The dataset employs the 2000 Commuting Zone ID, covering all 48 states, the District of Columbia, and Hawaii. We utilize the county-commuting zone crosswalk provided by the USDA for mapping purposes.

Within the Lightcast dataset, multiple variables define each vacancy's occupation, including the SOC code, standardized job title, and Lightcast occupation. Our focus for labor market concentration data centers on the six-digit SOC. In our study, the SOC codes assigned to jobs were derived using Lightcast occupation coding rules. Specifically, SOC codes were extracted from the first six digits of each job's O*NET code. It is important to note that our analysis relies on SOC codes based on the most recent 2010 SOC delineations. For example, the SOC code ”15-1199” corresponds to the occupation “Computer Occupations, All Other.”

The original Lightcast sample consisted of 431,422 markets with 903 commuting zones and 836 six-digit SOC. This leads to a total of 9,308,224 observations by commuting zone-SOC, and year-quarter (market-period level data).

## 4 Experimental Design, Materials and Methods

The raw year-month job posting data was provided from Lightcast as a text file on July 12, 2021. To import the raw data into STATA, we use the dictionary files provided by Lightcast. First, we import, create, and save employer and job title ID number. Then, we import and keep information on job ID, job posting date, occupation (six-digit SOC), county fips, employer name, education required, salary, and work hours. We merge these variables with employer and job title ID number and append through all years *(Import data_employer number, job title (11.30.23).do file).*

To calculate labor market concentration, we import the appended data and drop observations with missing counties and SOC information. Then, we use county-commuting zone crosswalk provided by the USDA which is available at https://www.ers.usda.gov/data-products/commuting-zones-and-labor-market-areas/. We keep the county FIPS and 2000 Commuting Zone ID and save it as “*county_cz_xwalk.dta*” which is available in our replication package. The USDA's commuting zone delineations have not been updated since the 2000 census. However, more recent commuting zones based on the 2010 census are available and can be found on the Penn State website under “Labor-sheds for Regional Analysis” [6]. To map county data after 2000 to the 2000 commuting zones, researchers need to make adjustments for rare cases of county splits, mergers, or changes in FIPS codes resulting from name changes or administrative adjustments that occurred in the 2010s and beyond. However, it's important to note that this study did not address these specific data adjustments.

We define labor market concentration as the Herfindahl-Hirschman Index (HHI) at the six-digit SOC occupation by commuting zone by quarter level. For the HHI based on vacancies, the market share of a firm in a given market and year-quarter is defined as the sum of vacancies posted in Lightcast by a given firm in a given market and year-quarter divided by total vacancies posted on the website in that market and year-quarter. To calculate the lower bound of HHI, we assume that all the missing employer names are different from one another and from postings by identified firms, thus providing a lower bound for labor market concentration. We also calculate the higher bound of HHI, assuming that missing employer names represent one single firm. The formula for the HHI is:

$$HHI_{m,t}=\sum_{j=1}^{J}S_{j,m,t}^{2}$$

where $S_{j,m,t}$ is the market share of firm *j* in market *m*. Using this formula, we measure HHI and multiply HHI, which is originally between 0 and 1, by 10,000. This scaling enhances readability and aligns with official merger guidelines. In antitrust practice as outlined in the Department of Justice/Federal Trade Commission 2010 horizontal merger guidelines, a market is deemed highly concentrated if its HHI exceeds 2500, and it is considered moderately concentrated when the HHI falls between 1500 and 2500 [2]. For a detailed breakdown of each step, refer to the *Calculate HHI (11.30.23).do* file.

## Limitations

The limitation of Lightcast data is its exclusive coverage of jobs posted on online platforms, leading to a potential over-representation of higher-skilled occupations and industries. Notably, job postings on Lightcast exhibit a bias towards more skilled occupations [5]. However, a comparison of Lightcast data with official employment data, such as the U.S. Occupational Employment Statistics and the Job Opening and Labor Turnover Survey (JOLTS), reveals good representativeness at both the occupational and industry levels [4,5]. Also, 30–40 % of employer names is missing, often attributed to staffing companies not disclosing the entities on whose behalf they post jobs [2]. To address this issue, we calculate both the lower and upper bounds of the HHI as discussed in the methods. Furthermore, in the original Lightcast data, the employer variable has been standardized, grouping variants of employer names together. For instance, postings from “Burning Glass,” “Burning Glass Technologies,” and “Burning Glass International, Inc.” are all standardized to “Burning Glass Technologies.” However, this approach has limitations as it may fail to differentiate between different employers with the same name. It also fails to identify employers with different names as the same employer, when employers with different names operate under the same ownership. Finally, it's crucial to note that Lightcast data is subject to constant updates. If researchers obtain Lighcast data after or before our data download period, the results may vary, and exact replication of the data may not be possible.

## Ethics Statement

The authors have read and followed the ethical requirements for publication in Data in Brief. We confirm that the current work does not involve human subjects, animal experiments, or any data collected from social media platforms.

## CRediT authorship contribution statement

**Hyeri Choi:** Methodology, Software, Formal analysis, Visualization. **Ioana Marinescu:** Methodology, Software, Formal analysis, Visualization.

## Data Availability

Data for labor market concentration using Lightcast (formerly Burning Glass Technologies) (Original data) (Mendeley Data).
